# Next‐Generation Immune Checkpoints and Tumor Microenvironment Modulation in Cancer Immunotherapy

**DOI:** 10.1155/jimr/7864229

**Published:** 2026-02-16

**Authors:** Jhommara Bautista, Carolina E. Echeverría, Iván Maldonado-Noboa, Joseth Adatty-Molina, Salomé Suárez Urresta, Emily C. Coral-Riofrio, Salomé Araujo-Abad, Nikolaos C. Kyriakidis, Andrés López-Cortés

**Affiliations:** ^1^ Cancer Research Group (CRG), Faculty of Medicine, Universidad de Las Américas, Quito, Ecuador, udla.edu.ec; ^2^ Department of Medicine, New York University Grossman School of Medicine, New York, USA, med.nyu.edu; ^3^ Jefe de servicio de oncología, Hospital Metropolitano de Quito, Quito, Ecuador; ^4^ Hospital General Universitario Dr. Balmis, Alicante, Spain; ^5^ Cancer Research Group, Faculty of Engineering and Applied Sciences, Universidad de Las Américas, Quito, Ecuador, udla.edu.ec; ^6^ Center for Hematology and Regenerative Medicine, Department of Medicine Huddinge, Karolinska Institute, Stockholm, Sweden, ki.se

## Abstract

Immunotherapy has reshaped the oncology landscape by enabling the immune system to recognize and eliminate malignant cells. Although immune checkpoint inhibitors (ICIs) targeting cytotoxic T‐lymphocyte‐associated protein 4 (CTLA‐4), programmed cell death‐1 (PD‐1), and programmed death‐ligand 1 (PD‐L1) have achieved durable responses in several cancers, their therapeutic benefit remains limited to a subset of patients, largely due to immune evasion, tumor heterogeneity, and immunosuppressive features of the tumor microenvironment (TME). This review comprehensively examines the expanding landscape of next‐generation immune checkpoints, encompassing both co‐inhibitory (lymphocyte activation gene‐3 [LAG‐3], T cell immunoglobulin and mucin‐domain containing‐3 [TIM‐3], TIGIT, VISTA, and IGSF8) and co‐stimulatory (ICOS, OX40, GITR, 4‐1BB, CD40, and CD27) pathways that collectively regulate the balance between immune activation and tolerance. We discuss their molecular mechanisms, translational rationale, and emerging clinical evidence, highlighting their potential to reinvigorate antitumor immunity, particularly in ICI‐refractory settings. Beyond checkpoint modulation, we explore complementary strategies aimed at remodeling the TME and enhancing immune responsiveness, including targeting immunometabolic pathways (IDO1 and CD73), innate immune sensing (toll‐like receptors [TLRs]), cytokine signaling (IL‐2), micronutrient immunoregulators (vitamin D), and the gut microbiota. The integration of these approaches into rational combination regimens, guided by predictive features such as T cell infiltration, tumor mutational burden (TMB), and microbiome composition, holds promise for extending the clinical impact of immunotherapy across malignancies. We further advocate for a precision immuno‐oncology framework that leverages multiomic profiling, systems biology, and artificial intelligence (AI) to optimize therapeutic selection and mitigate immune‐related toxicities. Emerging advances in nanomedicine, synthetic biology, and chronotherapy offer additional opportunities to enhance therapeutic specificity and durability, collectively charting a path from mechanistic insight to clinical translation toward realizing the full curative potential of cancer immunotherapy.

**Trial Registration:** ClinicalTrials.gov identifier: LAG‐3 (NCT03470922, NCT04082364, NCT05064059, NCT05352672, NCT02614833, NCT03625323, NCT01968109), TIM‐3 (NCT03307785, NCT03680508, NCT02608268, NCT02817633), TIGIT (NCT03563716), VISTA (NCT02671955, NCT02812875), IGSF8 (NCT05669430), CD73 (NCT02503774), B7‐H3 (NCT02475213, NCT01391143, NCT02628535, NCT03406949, NCT00089245, NCT01099644, NCT01502917), OX40 (NCT01862900, NCT02315066, NCT02410512, NCT02221960, NCT02528357, NCT02923349, NCT02705482), CD27 (NCT02335918, NCT02924038, NCT02302339, NCT02386111, NCT02543645), 4‐1BB (NCT01307267, NCT02444793, NCT01471210, NCT02253992, NCT02554812), CD40 (NCT02588443, NCT03329950), ICOS (NCT02904226, NCT02723955, NCT03251924), GITR (NCT02583165, NCT02132754, NCT02697591, NCT03126110, NCT02740270, NCT02598960, NCT01239134, NCT02628574), IDO1 (NCT02752074, NCT02658890, NCT02077881, NCT01560923, NCT02073123, NCT02327078, NCT02178722), TLRs (NCT02556463, NCT02042781), and IL‐2–based therapies (NCT02869295, NCT02983045)

## 1. Introduction

Immunotherapy has rewired the oncology landscape by enabling the immune system to selectively destroy malignant cells [1]. At the forefront of this transformation are immune checkpoint inhibitors (ICIs), particularly those targeting cytotoxic T‐lymphocyte‐associated protein 4 (CTLA‐4), the programmed cell death‐1 (PD‐1), and the programmed death‐ligand 1 (PD‐L1). These therapies have achieved durable clinical responses and improved survival in melanoma, renal cell carcinoma (RCC), and non‐small cell lung cancer (NSCLC) [2–4]. Nevertheless, benefits remain heterogeneous, as both intrinsic and acquired resistance continue to limit long‐term effectiveness. Resistance mechanisms are multifactorial and include tumor heterogeneity, immune evasion strategies, metabolic dysregulation such as aerobic glycolysis, and an immunosuppressive tumor microenvironment (TME) that collectively impair effective immune surveillance [5–8].

In light of these challenges, the focus of immuno‐oncology has expanded beyond CTLA‐4, PD‐1, and PD‐L1 to encompass a broader array of regulatory pathways [3, 4, 9]. The immune system relies on a sophisticated array of checkpoint molecules that modulate T cell behavior by balancing activation and suppression signals [10]. Several newly characterized co‐inhibitory targets, such as lymphocyte activation gene‐3 (LAG‐3) [11], T cell immunoglobulin and mucin‐domain containing‐3 (TIM‐3) [12, 13], TIGIT [14], VISTA [15, 16], Siglec‐15 [17], IGSF8 [18, 19], CD73 [20], BTLA/CD272, and B7‐H3 [21], have emerged as potential regulators of immune suppression within the TME. These pathways contribute to T cell dysfunction, promote regulatory T cell (Treg) activity, and shape myeloid cell‐mediated immunosuppression, facilitating tumor immune escape [22]. Blocking these inhibitory receptors, either alone or in combination with established ICIs, is under active investigation for their potential to reinvigorate antitumor immunity.

Importantly, the clinical relevance of immune checkpoints extends far beyond melanoma, NSCLC, and RCC. In gastric cancer, PD‐1 inhibitors such as nivolumab and pembrolizumab have become integral components of first‐line and second‐line therapy, particularly in PD‐L1–positive or microsatellite instability (MSI)‐high tumors, producing significant survival benefits [23–25]. In prostate cancer, traditionally considered immunologically “cold,” ICIs have shown activity in subsets of patients with MSI, *CDK12* alterations, or high tumor mutational burden (TMB), and combination strategies with PARP inhibitors or hormonal therapy are rapidly advancing [26, 27]. Ovarian cancer, despite historically modest responses to PD‐1/PD‐L1 blockade, demonstrates emerging sensitivity when ICIs are paired with anti‐angiogenic agents, PARP inhibitors, or myeloid‐targeting therapies [28, 29]. In colorectal cancer, durable responses to ICIs in MSI‐H/dMMR tumors represent one of the most compelling examples of precision immunotherapy, whereas microsatellite stability (MSS) tumors require novel combinatorial strategies [30, 31]. Additionally, pancreatic, biliary, and glioblastoma tumors, long considered refractory, exhibit actionable immune‐suppressive niches, and next‐generation checkpoints are entering early clinical evaluation to overcome profound T cell exclusion and myeloid‐dominant immunosuppression [32, 33].

Concurrently, the regulation of co‐stimulatory receptors is being explored as a complementary means to enhance antitumor immunity. Receptors such as ICOS [34], OX40 [35], GITR [36], 4‐1BB [37], CD40 [38], and CD27 [39] promote T cell proliferation, cytokine production, and survival. Early‐phase clinical trial data indicate that agonists targeting these pathways may potentiate responses to ICIs while maintaining a tolerable safety profile [22, 40]. This review provides an in‐depth examination of next‐generation immune checkpoint pathways and TME–modulating strategies. We explore the molecular mechanisms underpinning co‐inhibitory and co‐stimulatory signaling, evaluate recent clinical advances, and discuss ongoing translational challenges that must be addressed to broaden the therapeutic benefit of immunotherapy across diverse cancer types.

## 2. Co‐Inhibitory Targets in Immunotherapy

**Figure 1:**
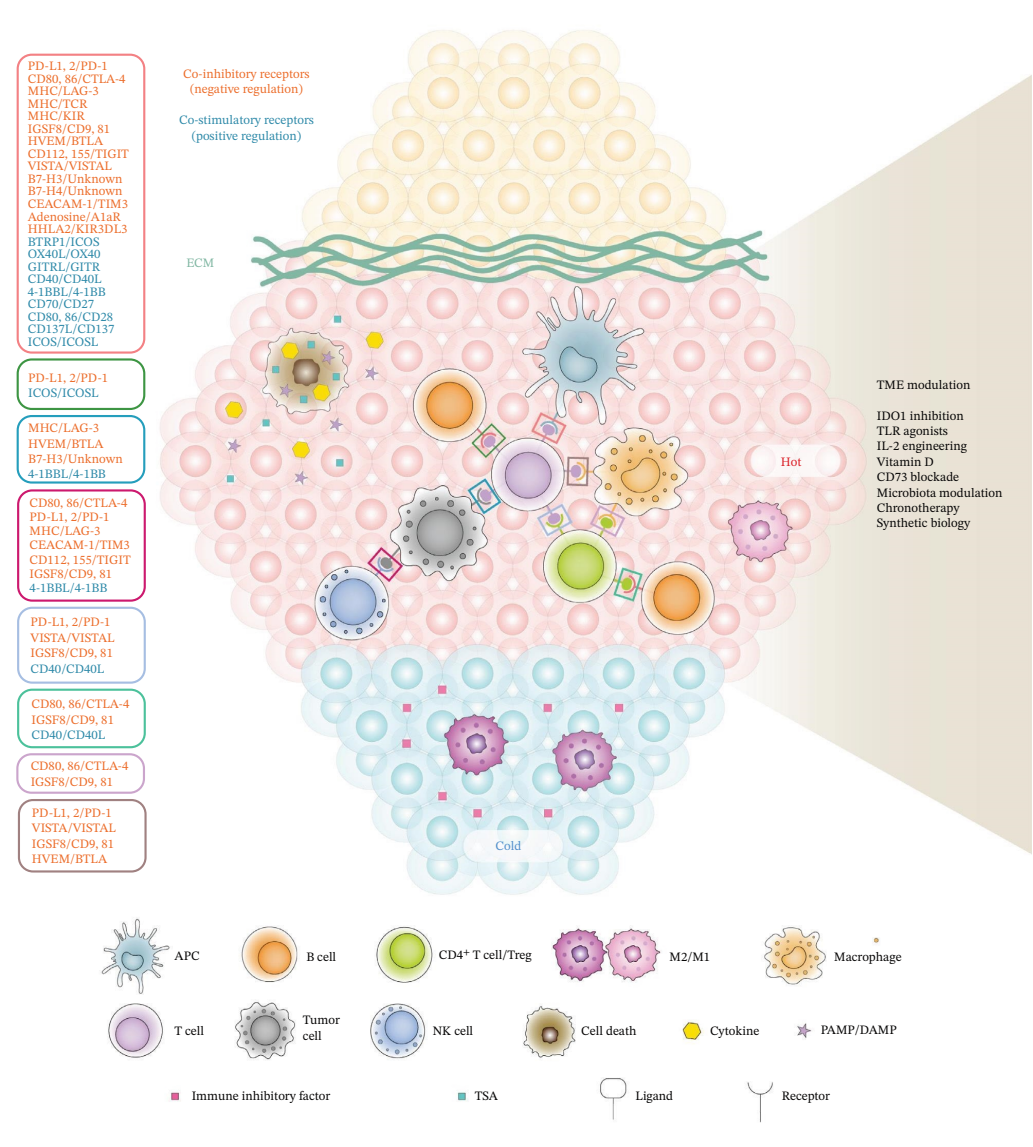
Key cellular players and regulatory interactions in the tumor immune response. Tumor immunity is orchestrated through the recognition of tumor‐associated antigens by antigen‐presenting cells (APCs), which activate T cells via antigen presentation and co‐stimulatory signals. Effective antitumor responses require a balance between activating and inhibitory immune checkpoints. In addition to T cells, other immune components such as B cells, natural killer (NK) cells, and macrophages engage with tumor and stromal elements through diverse ligand–receptor interactions that shape the immune landscape of the tumor microenvironment.

Beyond CTLA‐4 and PD‐1/PD‐L1, several other immunosuppressive receptors with negative regulation like CD73, LAG‐3, TIM‐3, BTLA, TIGIT, VISTA, Siglec‐15, IGSF8, and B7‐H3 represent promising avenues to improve immunological treatments and overcome resistance (Figure 1 and Table 1). Collectively, these checkpoints form an interconnected suppressive network within the TME, and their coordinated blockade is increasingly viewed as essential for next‐generation immunotherapy.

**Table 1:** Chronological landscape of next‐generation inhibitory immune checkpoints in cancer immunotherapy.

Checkpoint	Type	Year (discovery/first‐in‐human)	Ligands/binding partners	Mechanism of action	Therapeutic agents	Clinical stage	Cancer types
CTLA‐4	Inhibitory	1987/2000	CD80, CD86	Competes with CD28; performs trans‐endocytosis depleting CD80/86; boosts Tregs; induces IDO	Ipilimumab, tremelimumab	FDA–approved	Melanoma, RCC, CRC, HCC
PD‐1	Inhibitory	1992/2006	PD‐L1, PD‐L2	Inhibits PI3K/AKT and MAPK signaling; induces exhaustion; suppresses cytotoxicity/proliferation	Pembrolizumab, nivolumab, cemiplimab	FDA–approved	NSCLC, melanoma, RCC, gastric, CRC, TNBC
CD73	Inhibitory (metabolic)	1990s/2015	AMP–adenosine	Produces immunosuppressive adenosine; inhibits CD8/NK; stabilizes Tregs	Oleclumab, NZV930	Phase 1–2	NSCLC, breast, pancreatic
LAG‐3	Inhibitory	1990/2015	MHC‐II, galectin‐3	High‐affinity MHC‐II binding inhibits CD4^+^; enhances Tregs; maintains exhausted T cells	Relatlimab	FDA–approved 2022	Melanoma, NSCLC, CRC
TIM‐3	Inhibitory	2002/2015	Galectin‐9, CEACAM1, HMGB1, PtdSer	Drives exhaustion; suppresses DC nucleic acid sensing; impairs NK cytotoxicity	Sabatolimab, cobolimab	Phase 2–3	AML, MDS, NSCLC
BTLA	Inhibitory	2003/2015	HVEM	Inhibits TCR signaling; expands Tregs; reduces CD8 cytotoxicity	TAB004	Preclinical Phase 1	Lymphoma, melanoma, gastric
TIGIT	Inhibitory	2009/2016	CD155, CD112	Recruits SHP2; suppresses T/NK cytotoxicity; competes with CD226	Tiragolumab, vibostolimab	Phase 3	NSCLC, CRC, gastric
VISTA	Inhibitory	2011/2016	VSIG‐3	Myeloid‐driven T cell suppression; cytokine inhibition; PD‐1‐independent	CA‐170, JNJ‐61610588	Phase 1	Pancreatic, NSCLC, prostate
Siglec‐15	Inhibitory	2019/2020	Sialylated glycans	Suppresses antigen‐specific T cells; maintains immune‐cold states; mutually exclusive with PD‐L1	NC318	Phase 1–2	NSCLC, HNSCC, bladder
IGSF8	Inhibitory	2001 (redefined 2023)/2023	Unknown	Regulates β‐catenin degradation; suppresses antigen presentation; myeloid‐mediated immunosuppression	GV20‐0251	Phase 1	Lung, bladder, CRC, endometrial
B7‐H3 (CD276)	Inhibitory	2001/2013	Unknown	NK inhibition; promotes angiogenesis, metastasis, glycolysis	Enoblituzumab, DS‐7300	Phase 1–2	Prostate, neuroblastoma, NSCLC

*Note:* CEACAM1, carcinoembryonic antigen–related cell adhesion molecule 1; NK, natural killer cell; PD‐1, programmed cell death protein 1; PD‐L1/PD‐L2, programmed death ligand 1/2; VSIG‐3, V‐set and immunoglobulin domain containing 3.

Abbreviations: AML, acute myeloid leukemia; AMP, adenosine monophosphate; CD, cluster of differentiation; CRC, colorectal cancer; CTLA‐4, cytotoxic T‐lymphocyte antigen 4; DC, dendritic cell; HCC, hepatocellular carcinoma; HMGB1, high‐mobility group box 1; HNSCC, head and neck squamous cell carcinoma; HVEM, herpesvirus entry mediator; IDO, indoleamine 2,3‐dioxygenase; IGSF8, immunoglobulin superfamily member 8; MDS, myelodysplastic syndromes; MHC‐II, major histocompatibility complex Class II; NSCLC, non‐small cell lung cancer; PtdSer, phosphatidylserine; RCC, renal cell carcinoma; TCR, T cell receptor; TNBC, triple‐negative breast cancer; Treg, regulatory T cell.

### 2.1. CTLA‐4

CTLA‐4 was the first target for clinical immunotherapy trials because of its ability to attenuate T cell activation [3, 4]. It encodes an inhibitory receptor that competes with CD28 for binding to the CD80 (B7.1) and CD86 (B7.2) co‐stimulatory ligands on antigen‐presenting cells (APCs), exhibiting roughly 100‐fold higher binding affinity [41]. Following TCR engagement, CTLA‐4 is mobilized from internal cell stores to the surface, where it downregulates immune responses by inhibiting co‐stimulation [42–44]. CTLA‐4 suppresses immune activation through both intrinsic and extrinsic pathways. Beyond competing with CD28, CTLA‐4 also facilitates the trans‐endocytosis (or trogocytosis) of CD80/CD86 ligands from APCs, an immunoregulatory mechanism primarily executed by Tregs, which depletes ligands necessary for effective T cell activation at the immunological synapse [3]. Additional immunosuppressive roles of CTLA‐4 include direct inhibitory signaling, suppression of CD8^+^ T cell activity [45], modulation of T helper (Th) cell differentiation [46], activation of the immunosuppressive enzyme IDO [47], and interference with protein trafficking pathways [48].

The therapeutic quality of CTLA‐4 blockade was established by James Allison’s seminal work, which showed that inhibiting CTLA‐4 could reinvigorate antitumor T cell responses [49–51]. This discovery catalyzed the creation of fully human monoclonal antibodies (mAbs) such as ipilimumab and tremelimumab [49, 50]. Ipilimumab became the first CTLA‐4 inhibitor to receive U.S. Food and Drug Administration (FDA) approval in 2011 for metastatic melanoma and has been incorporated into combination protocols for RCC and MSI‐H metastatic colorectal cancer (mCRC) [52, 53]. Tremelimumab is an IgG2 mAb that was approved in 2022 in combination with durvalumab for hepatocellular carcinoma (HCC), with ongoing investigations into its efficacy in additional malignancies [53–55]. Although CTLA‐4 inhibitors have shown durable clinical responses, the proportion of patients who benefit remains modest (around 15%), and treatment is linked with immune‐related adverse events (irAEs) [56, 57]. Nonetheless, some individuals experience long‐term remission, occasionally lasting more than a decade, prompting the incorporation of OS as a key end point in clinical trials [58–60]. To improve clinical outcomes and reduce toxicity, novel CTLA‐4‐targeting agents are under investigation, including erfonrilimab, quavonlimab, cadonlimab, and zalifrelimab. Emerging data suggest that patients with tumors harboring high TMB may derive greater benefit from CTLA‐4 blockade, providing a rationale for more personalized immunotherapy strategies [61, 62].

### 2.2. PD‐1 and Its Ligands

The PD‐1 (CD279) signaling pathway preserves immune homeostasis by modulating peripheral tolerance and preventing autoimmunity during inflammation [9, 10, 22, 63]. PD‐1, encoded by *PDCD1*, is a Type I transmembrane glycoprotein structured by an extracellular immunoglobulin V‐like (IgV) region, a transmembrane region, and a cytosolic tail with ITIMs [64]. PD‐1 is predominantly expressed on activated natural killer (NK) cells, T cells, B cells, and various myeloid lineages [40, 65]. Unlike CTLA‐4, which modulates early T cell activation primarily within lymphoid tissues, PD‐1 is enrolled in peripheral tissues to restrain excessive immune responses. Within the TME, PD‐1 signaling contributes to tumor immune evasion by inhibiting Teff cell activity [66, 67]. Upon engagement with the PD‐L1/CD274/B7‐H1 and PD‐L2/CD273/B7‐DC ligands, PD‐1 suppresses downstream signaling like PI3K/AKT and Ras/MEK/ERK, leading to reduced T cell proliferation, cytotoxic function, and cytokine production. Moreover, PD‐L1 is broadly expressed by malignant cells and other nonimmune components of the TME, exacerbating local immunosuppression [68]. The heterogeneous expression patterns of PD‐L1 and PD‐L2, particularly on tumor‐infiltrating lymphocytes (TILs), are identified as key contributors to immune escape mechanisms in cancer [68].

The clinical relevance of PD‐1 blockade was validated in 2014 with the FDA approval of pembrolizumab, a humanized mAb targeting PD‐1 to treat advanced melanoma and NSCLC [69]. This milestone was soon complemented by the emergence and approval of additional anti‐PD‐1 agents (nivolumab, cemiplimab, and dostarlimab) and anti‐PD‐L1 agents (atezolizumab, avelumab, and durvalumab) [70]. These inhibitors have demonstrated long‐lasting clinical benefits and favorable safety profiles across diverse malignancies, including triple‐negative breast cancer (TNBC), RCC, CRC, urothelial carcinoma, and Merkel cell carcinoma [63, 71–78]. Despite their success, PD‐1/PD‐L1 inhibitors are affected with irAEs, which may affect multiple organ systems and manifest as endocrinopathies, pneumonitis, hepatitis, colitis, dermatologic reactions, and, in rare cases, Type 1 diabetes [40, 79]. To mitigate toxicity while improving efficacy, a new generation of PD‐1 and PD‐L1 inhibitors is under clinical evaluation. Among the PD‐1‐targeting agents are retifanlimab‐dlwr, tislelizumab, sintilimab, toripalimab, zimberelimab, spartalizumab, codonilimab, and others, while novel PD‐L1 blockers include sugemalimab, bintrafusp alfa, pacmilimab, and danburstotug [62]. These candidates aim to refine immune modulation through enhanced selectivity, improved pharmacokinetics, or reduced immune toxicity. In addition to drug development, identifying reliable biomarkers for patient selection remains a key focus. TMB has emerged as a predictor biomarker, with higher TMB levels correlating with increased responsiveness to PD‐1/PD‐L1 blockade [6].

### 2.3. LAG‐3

The inhibitory receptor LAG‐3 (CD223) is upregulated in multiple cells like Tregs, CD4^+^ and CD8^+^ T cells, B cells, NK cells, and DCs [11]. Despite sharing structural similarities with CD4, LAG‐3 demonstrates significantly greater attraction for major histocompatibility complex Class II (MHC‐II) molecules, which enables it to serve as a potent negative regulator of immune activation [80, 81]. Through interaction with MHC‐II on APCs, LAG‐3 downregulates CD4^+^ T cell proliferation, impairs cytotoxic CD8^+^ T cell responses, and enhances the suppressive activity of Tregs [82, 83]. These immunosuppressive effects are exploited within the TME, where LAG‐3 contributes to immune escape by maintaining a state of T cell exhaustion and dampening antitumor immunity [84].

Therapeutic application of LAG‐3 has recently transitioned from preclinical exploration to clinical application. The FDA approved the combination therapy between nivolumab and relatlimab‐rmbw, an anti‐LAG‐3 mAb, in 2022 [85]. This fixed‐dose regimen, marketed as Opdualag, demonstrated an important improvement in progression‐free survival (PFS) in individuals with unresectable or metastatic melanoma, extending median PFS from 4.6 months with nivolumab monotherapy to 10.1 months with the combination [86]. However, a higher rate of Grade ≥3 adverse events was observed with combination therapy (18.9% vs. 9.7%), underscoring the importance of careful toxicity management [86]. Beyond relatlimab, several LAG‐3‐targeting agents are undergoing clinical evaluation, including MK‐4280, fianlimab, tebotelimab, eftilagimod alpha, and ieramilimab [14, 87]. As data from these studies mature, LAG‐3 may further solidify its role as a key therapeutic target in next‐generation immune checkpoint strategies.

### 2.4. TIM‐3

The inhibitory receptor TIM‐3 is increasingly recognized for its role in promoting tumor immune escape [12, 13]. It is regulated on multiple immune cells, including DCs, NK cells, Tregs, CD4^+^ and CD8^+^ T cells, and TILs, particularly in chronic inflammation and cancer. TIM‐3 exerts its immunosuppressive effects through interaction with multiple ligands like high‐mobility group box 1 (HMGB1), phosphatidylserine, carcinoembryonic antigen‐related cell adhesion molecule 1 (CEACAM‐1), and galectin‐9 [88]. These ligand–receptor interactions trigger the T cells dysfunction and exhaustion, thereby dampening antitumor immunity and facilitating immune evasion within the TME.

Preclinical studies have revealed that inhibition of TIM‐3 signaling restores Teff cell function, reverses T cell exhaustion, and enhances cytotoxic responses against malignant cells [89]. These findings have prompted the development of TIM‐3‐targeting therapies that are currently undergoing clinical trials, frequently in combination with PD‐1 inhibitors. Notable anti‐TIM‐3 agents under investigation include sabatolimab, cobolimab, and RO‐7121661, which are being assessed across a range of malignancies in early‐phase and ongoing clinical studies [62, 90]. Preliminary evidence from preclinical models and early clinical experience suggests that dual blockade of TIM‐3 and PD‐1 may provide enhanced therapeutic benefit, particularly in tumors characterized by high TIM‐3 expression and profound T cell exhaustion.

### 2.5. T Cell Immunoglobulin and Immunoreceptor Tyrosine‐Based Inhibitory Motif Domain

TIGIT is an emerging checkpoint target enrolled in dampening both T cell and NK cell responses [14]. It exerts its inhibitory effects through binding to poliovirus receptors (CD155) and nectin‐2 (CD112), regulated on APCs and tumor cells [91]. These interactions lead to immune cell suppression and promotion of immunoregulatory cytokine production, contributing to an immunosuppressive TME [14]. TIGIT is encompassed by an extracellular IgV region, a Type I transmembrane region, and an intracellular tail that contains both an ITIM and an immunoreceptor tyrosine‐based turn motif–like (ITT)–like motif [22, 92, 93]. Upon ligand binding, these motifs are phosphorylated, leading to recruitment of SHP phosphatases that inhibit downstream signaling pathways. This results in reduced proliferation of Teff cells and downregulation of IFN‐γ and IL‐17 pro‐inflammatory cytokines.

TIGIT is often found on dysfunctional CD8^+^ T cells in the TME, where it is co‐expressed with the PD‐1 and CTLA‐4 receptors [94, 95]. Experimental models have shown that TIGIT blockade reverses T cell exhaustion and enhances cytotoxic effector function, providing a strong mechanistic rationale for combinatorial checkpoint inhibition strategies. Consistent with this rationale, the combination of the anti‐TIGIT antibody tiragolumab with the anti‐PD‐L1 agent atezolizumab demonstrated superior clinical activity compared with atezolizumab monotherapy in a randomized Phase II study [96]. In metastatic NSCLC patients with upregulated PD‐L1, this combination increased the overall response rate from 21% to 37%, leading to the designation of tiragolumab as a breakthrough therapy by the FDA [90, 97].

To further enhance the effectiveness of TIGIT–targeted therapies, nanomedicine platforms are being developed to enable localized delivery within tumors, aiming to improve therapeutic index and reduce systemic adverse effects [98]. Additionally, dual immune checkpoint blockade involving TIGIT and TIM‐3 has shown synergistic effects by simultaneously interrupting multiple immunosuppressive circuits, yielding stronger antitumor responses in preclinical models [99, 100]. Consistently, meta‐analyses have linked elevated expression of TIGIT and TIM‐3 to poor clinical outcomes across multiple cancers, supporting their value as therapeutic targets and prognostic biomarkers [94, 101]. Several additional anti‐TIGIT mAbs are currently under clinical investigation, including vibostolimab, domvanalimab, ociperlimab, etigilimab, IBI‐939, and ASP‐8374 [62]. The focus on TIGIT highlights its emerging role in shaping future immunotherapeutic approaches designed to bypass treatment resistance and extend clinical benefit.

### 2.6. V‐Domain Ig‐Containing Suppressor of T Cell Activation

The inhibitory receptor VISTA or programmed death‐1 homolog (PD‐1H) belongs to the B7 family enrolled in modulating immune responses. Unlike many other checkpoint molecules that are upregulated upon activation, this receptor is expressed, primarily on myeloid‐derived cells like monocytes and DCs, and to a lesser extent on T cells and NK cells [15, 16]. Through its suppressive functions, VISTA contributes to peripheral immune tolerance by inhibiting T cell activation, limiting cytokine production, and reducing T cell proliferation [102]. VISTA’s involvement in tumor immune evasion has gained increasing attention. Upregulation of VISTA has been observed in multiple human cancers, where it contributes to the immunosuppressive TME and is frequently linked with poor clinical prognosis [103]. Interestingly, VISTA exerts its immunoregulatory effects even in malignant cells with high PD‐L1 expression or in cases where VISTA itself is not expressed by tumor cells, suggesting its functional independence from the PD‐1/PD‐L1 axis [104]. Experimental models have shown that blockade of VISTA enhances the activation of TILs and augments tumor‐specific T cell responses, supporting its role as a nonredundant target in immunotherapy.

Given that PD‐1 and VISTA operate through distinct regulatory mechanisms, dual checkpoint blockade has the potential to synergistically enhance antitumor immunity [105]. However, therapeutic targeting of VISTA must be approached with caution, as disruption of its immunoregulatory functions may precipitate irAEs due to loss of immune homeostasis. Several VISTA‐directed agents are currently undergoing early‐phase clinical evaluation. Among these, JNJ‐61610588 and CA‐170—an orally available small molecule that targets both VISTA and PD‐L1/PD‐L2—are being assessed in patients with advanced solid tumors to define the safety, efficacy, and immunomodulatory consequences of VISTA inhibition [22].

### 2.7. Siglec‐15

It has emerged as a nonredundant immune suppressor with a regulatory profile distinct from the PD‐1/PD‐L1 axis. It is primarily expressed on tumor‐associated macrophages (TAMs), M2‐polarized myeloid cells, osteoclast‐lineage cells, and a subset of tumor cells, while exhibiting minimal expression in most normal tissues. This restricted but tumor‐enriched distribution underscores its role in shaping an immunosuppressive microenvironment [17, 106, 107]. Mechanistically, Siglec‐15 recognizes sialylated glycans and delivers inhibitory signals that suppress antigen‐specific T cell activation, contributing to T cell dysfunction and maintaining “immune‐cold” tumor states [17]. Importantly, Siglec‐15 and PD‐L1 show largely mutually exclusive expression patterns across human cancers, suggesting that Siglec‐15 may drive immune escape in tumors that do not rely on PD‐L1 upregulation. This makes Siglec‐15a compelling therapeutic target for patients who fail to respond to PD‐1/PD‐L1 blockade [17]. Preclinical evidence shows that Siglec‐15 inhibition restores CD8^+^ T cell cytotoxicity and suppresses tumor growth even in PD‐1–refractory models. Early clinical evaluation of the first‐in‐class Siglec‐15 antibody NC318 has demonstrated initial signs of activity in NSCLC and other solid tumors [108, 109].

### 2.8. Immunoglobulin Superfamily Member 8

IGSF8 (EWI‐2, PGRL, or CD316) is a transmembrane glycoprotein initially characterized in neural tissues, where it is abundantly expressed at the plasma membrane [18, 19]. Recent research has repositioned IGSF8 as a novel immune checkpoint due to its emerging role in regulating antitumor immunity. Its expression profile varies across tumor types, being elevated in HCC, pancreatic cancer, and glioblastoma, but reduced in melanoma [110]. Functional studies have revealed that IGSF8 exerts immunosuppressive effects that promote tumor progression. Deletion of IGSF8 in melanoma models reduces tumor growth, while in myeloid leukemia, its knockout impairs leukemia stem cell survival by enhancing apoptosis and promoting degradation of β‐catenin, a key oncogenic driver [110, 111]. These findings highlight IGSF8’s role in tumor maintenance across both solid and hematologic malignancies. Therapeutically, the first‐in‐class mAb GV20‐0251, an Fc‐attenuated IgG1, has demonstrated promising antitumor activity in preclinical models. This agent enhances antigen presentation, promotes NK cell activation, and disrupts IGSF8–mediated immunosuppressive signaling. GV20‐0251 is currently undergoing early‐phase clinical evaluation in patients with advanced solid tumors, including lung, bladder, colorectal, endometrial, and head and neck cancers [18].

### 2.9. Other Immune Checkpoint Blockade Strategies

Multiple immune checkpoint molecules are currently under active evaluation as potential therapeutic targets. Among them, CD73 is an ectoenzyme involved in the production of immunosuppressive adenosine within the TME. By converting AMP into adenosine, CD73 suppresses effector immune cell function and promotes tumor immune evasion. Therapeutic inhibition of CD73 using agents such as oleclumab, aims to reduce adenosine‐mediated immunosuppression and restore antitumor immunity, and is being explored in ongoing clinical development programs [20]. BTLA (CD272) is another inhibitory receptor expressed on T cells and other immune subsets. Engagement of BTLA with its ligand, the herpesvirus entry mediator (HVEM), transduces inhibitory signals that dampen T cell activation and facilitate malignant immune escape. Therapeutic strategies targeting the BTLA–HVEM axis are under investigation as a means to restore immune responsiveness and overcome immune suppression within the TME [20]. Another promising immune checkpoint target is B7‐H3, which is broadly upregulated across multiple tumor types and has been strongly associated with immune evasion mechanisms. Therapeutic approaches targeting B7‐H3 include mAbs such as omburtamab (8H9) and enoblituzumab, as well as combination strategies incorporating enoblituzumab with PD‐1/PD‐L1 blockade. These agents are being evaluated across a range of clinical development programs, reflecting growing interest in B7‐H3 as a viable target for cancer immunotherapy [21, 112–114].

## 3. Co‐Stimulatory Targets in Immunotherapy

In contrast to immune system inhibitory pathways, the ICOS, OX40, GITR, 4‐1BB, CD40, and CD27 co‐stimulatory molecules enhance positive immune regulation against malignant cells. Targeting these co‐stimulatory molecules can amplify the effectiveness of existing immunotherapies, providing a synergistic approach to complement the blockade of inhibitory checkpoints [115] (Figure 1 and Table 2).

**Table 2:** Chronological landscape of next‐generation stimulatory immune checkpoints in cancer immunotherapy.

Checkpoint	Type	Year (discovery/first‐in‐human)	Ligands/binding partners	Mechanism of action	Therapeutic agents	Clinical stage	Cancer types
OX40 (CD134)	Stimulatory	1987/2004	OX40L	Promotes Teff expansion, survival, cytokines; reduces Treg suppression; enhances NK activity	BMS‐986178, MEDI6469	Phase 1–2	Melanoma, CRC, HNSCC
CD27	Stimulatory	1987/2002	CD70	Supports Teff differentiation and memory formation; reduces Tregs	Varlilumab	Phase 1–2	Lung, melanoma, RCC
4‐1BB (CD137)	Stimulatory	1989/2004	4‐1BBL	Activates NF‐κB/MAPK; enhances CD8/NK cytotoxicity; improves CAR‐T persistence	Urelumab, utomilumab	Phase 1–2	Melanoma, lymphoma, breast
CD40	Stimulatory	1993/2000	CD40L (CD154)	APC licensing; enhances antigen presentation; activates NF‐κB, PI3K/Akt, MAPK, JAK/STAT pathways; repolarizes macrophages	CDX‐1140, APX005M, selicrelumab	Phase 1–2 (multiple combinations with PD‐1)	Pancreatic cancer, B‐cell lymphomas, melanoma
ICOS	Stimulatory	1999/2015	ICOSL	Enhances Th1/Th17/Tfh; increases memory T cells; synergizes with CTLA‐4 blockade	Feladilimab, vopratelimab	Phase 1–2	Breast, NSCLC, endometrial
GITR	Stimulatory	1999/2004	GITRL	Enhances Teff survival and cytotoxicity; reduces Treg suppression	MK‐4166, AMG 228	Phase 1–2	Melanoma, RCC, ovarian

*Note:* CAR‐T, chimeric antigen receptor T cells; GITR, glucocorticoid‐induced TNFR‐related protein; ICOS, inducible T cell co‐stimulator; NF‐κB, nuclear factor kappa‐light‐chainenhancerof activated B cells; NK, natural killer cells.

Abbreviations: CRC, colorectal cancer; GITRL, GITR ligand; HNSCC, head and neck squamous cell carcinoma; ICOSL, ICOS ligand; NSCLC, non‐small cell lung cancer; RCC, renal cell carcinoma; Teff, effector T cells; Treg, regulatory T cells.

### 3.1. OX40

OX40 (CD134), a member of a tumor necrosis factor receptor (TNFR) superfamily, delivers potent co‐stimulatory signals to activated immune cells. It is primarily regulated on activated CD4^+^ and CD8^+^ T cells, Tregs, and with lower expression observed on nonlymphoid immune cells such as neutrophils, NK cells, and endothelial cells [116, 117]. Upon engagement with its ligand OX40L, expressed on APCs, OX40 triggers downstream signaling pathways that promote T cell expansion, survival, cytokine production, and memory formation, while also enhancing NK cell–mediated responses [118]. In cancer, OX40 is upregulated on TILs, where its expression has been associated with favorable clinical outcomes [119]. Unlike immunotherapies aimed at depleting suppressive immune populations, OX40‐targeted strategies focus on boosting the functional capacity of Teff cells within the TME. This has allowed the generation of several agonistic mAbs targeting OX40, including vonlerolizumab (MOXR0916), ivuxolimab (PF‐04518600), MEDI6383, MEDI0562, MEDI6469, INCAGN01949, and GSK3174998. These agents are ongoing early‐phase clinical trials, both as monotherapies and in combinational therapies with other immunotherapeutic agents [22, 120–122]. Preliminary results from these studies are promising, with some agents showing immune activation and manageable safety profiles. However, in‐depth analysis is required to fully understand the biological process by which OX40 enhances antitumor immunity and to optimize its clinical application [123]. Continued translational and clinical investigations are critical for advancing OX40‐based therapies in immuno‐oncology.

### 3.2. Cluster of Differentiation 27

CD27, another TNFR superfamily member, is a co‐stimulatory protein critical for initiating and maintaining robust T cell responses [39]. It interacts with CD70, which is transiently expressed on activated T cells, B cells, and DCs. This function is essential for generating effector and memory T cells capable of mounting effective recall responses. Expression of CD27 and CD70 varies across cancers and has been linked to both favorable and unfavorable prognosis depending on the immune context of the TME. Therapeutic strategies have focused on agonistic antibodies such as varlilumab, which has shown potential in experimental models and early clinical studies. Varlilumab is under evaluation in trials combining it with checkpoint inhibitors like nivolumab and atezolizumab, demonstrating enhanced T cell infiltration and acceptable safety [124, 125].

Mechanistic studies reveal that CD27 agonism increases Teff cell proliferation, decreases Tregs, and promotes NK cell activation. For example, varlilumab treatment in lung adenocarcinoma reduced Treg frequencies while expanding cytotoxic T cells [126]. In murine models of melanoma, CD27 stimulation improved CD8^+^ T cell recruitment and enhanced antitumor immunity [127]. Combination therapies involving CD27 agonists and PD‐1/PD‐L1 inhibitors activate unique transcriptional programs in CD8^+^ T cells, enhancing efficacy without exacerbating immune‐related toxicity [128, 129]. Additionally, CD27 engagement has been shown to improve the efficacy of chimeric antigen receptor (CAR) T cells by modulating the immunosuppressive TME [130]. Nevertheless, CD27 signaling may exhibit dual roles depending on the immunologic context. While it often supports Teff function, some evidence suggests it can also sustain Treg populations under specific conditions, potentially limiting therapeutic efficacy [131]. Understanding this duality is key to optimizing CD27–based therapies.

### 3.3. 4‐1BB

4‐1BB (TNFRSF9 or CD137) is another co‐stimulatory protein within the TNFR superfamily that is expressed on T cells, NK cells, DCs, and other APCs [132]. Its interaction with 4‐1BB ligand (4–1BBL/CD137L) activates the NF‐κB, PI3K/Akt, and MAPK signaling pathways, which enhance T and NK cell proliferation, survival, and cytotoxicity [37]. 4‐1BB has also been successfully integrated into the design of CAR T cells to promote persistence and functionality [133]. Although early clinical trials were hindered by toxicity, particularly hepatotoxicity, next‐generation 4‐1BB agonists have been optimized for improved safety. Among these, utomilumab (PF‐05082566) is under evaluation alone and alongside other immunotherapeutics such as pembrolizumab, rituximab, mogamulizumab, OX40 agonists, and avelumab [134]. Another candidate, urelumab, is being evaluated in solid tumors and B cell non‐Hodgkin lymphoma, with ongoing efforts to balance efficacy and toxicity [132, 135].

### 3.4. Cluster of Differentiation 40

CD40 is a key receptor in the TNFR superfamily, primarily expressed on APCs and B cells. Its ligand, CD40L (CD154), is found on CD4^+^ T cells, activated T cells, and platelets [38]. The CD40–CD40L axis orchestrates both humoral and cellular immunity by stimulating cytokine production, B cell activation, and T cell priming, while also influencing thrombosis, hematopoiesis, and inflammation. Upon ligand engagement, CD40 trimerizes and recruits TNF receptor–associated factors (TRAFs), activating NF‐κB, PI3K/Akt, MAPK, and JAK/STAT downstream pathways. These signaling modulate immune cell polarization and remodel the TME by activating macrophages and promoting antigen presentation [136]. Multiple CD40 agonists, such as CDX‐1140, dacetuzumab, giloralimab, lucatumumab, selicrelumab, and sotigalimab, are in clinical development across a range of malignancies [137–142]. Selicrelumab has demonstrated particular promise in pancreatic cancer when combined with PD‐1 blockade, improving tumor‐specific T cell activity and clinical responses [143, 144]. Other agents like dacetuzumab and ABBV‐927 have shown preclinical efficacy in both solid tumors and B cell malignancies [145]. However, systemic CD40 activation can induce cytokine release syndrome (CRS) and thromboembolic complications, necessitating careful dose titration [146]. Research continues to identify optimal combination strategies and patient populations most likely to benefit [147].

### 3.5. Inducible T Cell Co‐Stimulator

The Type I transmembrane glycoprotein ICOS is part of the CD28/CTLA‐4 immune regulatory proteins [34]. Expressed primarily on activated T cells, ICOS is enrolled in modulating immune signals by promoting T cell proliferation, survival, and cytokine secretion. Its ligand, ICOSL, is found on APCs, and their interaction is relevant for the activation of Th 1, Th2, Th17, and Tregs. The ICOS–ICOSL signaling axis contributes to the expansion and functional specialization of both effector and Treg populations. Notably, ICOS expression is upregulated following anti‐CTLA‐4 treatment, suggesting its potential utility as a pharmacodynamic biomarker for checkpoint blockade response [148]. Additionally, ICOS triggers humoral immunity by facilitating the activation of follicular helper T (Tfh) cells, which in turn support B cell function and antibody production, an aspect relevant for cancer vaccine strategies [149]. ICOS also maintains memory T cell pools, which is essential for durable immune surveillance and long‐term tumor control. Given its multifaceted immunological functions, ICOS has become a therapeutic target in both oncology and autoimmune diseases [150]. In cancer, agonistic targeting of ICOS enhances immune responses. Preclinical melanoma models have shown that ICOS agonists promote tumor regression and synergize with PD‐1 blockade, highlighting their combination potential [151, 152]. Conversely, ICOS inhibition may be beneficial in autoimmune or allergic settings where immunosuppression is desirable.

Several ICOS‐targeting mAbs are being evaluated in clinical trials. Vopratelimab (JTX‐2011), an agonist anti‐ICOS IgG1 antibody, is under investigation in the ICONIC trial across various advanced malignancies including breast, lung, colorectal, pancreatic, and endometrial cancers. Interim results indicate a safety profile without dose‐limiting toxicities, though efficacy outcomes are pending [22, 153]. Another ICOS agonist, feladilimab (GSK3359609), an IgG4 mAb, is being assessed in the INDUCE‐1 trial as monotherapy and in combinational therapy with pembrolizumab for solid tumors and multiple myeloma [62, 151]. Importantly, combining ICOS stimulation with CTLA‐4 inhibition may offer enhanced efficacy, as CTLA‐4 blockade increases ICOS expression on Teff cells, thereby providing a mechanistic rationale for dual targeting approaches. Ongoing clinical studies are exploring such strategies, aiming to maximize therapeutic synergy and improve outcomes in immunotherapy‐resistant tumors.

### 3.6. Glucocorticoid‐Induced TNF Receptor‐Related Protein

GITR (TNFRSF18) is a member of the TNFR superfamily expressed on activated T cells, Tregs, and NK cells. Its ligand, GITRL, is predominantly present on DCs and macrophages [36, 154, 155]. Engagement of GITR by GITRL supports T cell survival by reducing apoptosis, promotes leukocyte adhesion, and enhances immune cell migration. In the TME, the GITR–GITRL axis is often exploited to suppress antitumor immunity. Agonistic antibodies targeting GITR mimic the ligand’s action by enhancing Teff function and attenuating Treg‐mediated suppression [156]. Multiple early‐phase clinical trials are currently investigating GITR agonists, including ragifilimab, TRX‐518, BMS‐986156, MEDI1873, MK‐4166, INCAGN01876, and GWN323, across a variety of solid and hematologic cancers [157, 158].

## 4. TME–Targeted Strategies

The TME serves as a critical modulator of cancer progression and immune escape, consisting of a dynamic interplay between tumor cells, stromal components, and infiltrating immune cells. Targeting the immunosuppressive mechanisms within the TME has become a pivotal focus in cancer immunotherapy, aiming to restore effective antitumor immune responses and improve the efficacy of current treatments. Key strategies under investigation include modulation of immunometabolism (e.g., IDO1 inhibition), innate immune sensing (e.g., toll‐like receptor [TLR] activation), and nutrient signaling (e.g., vitamin D), each offering a distinct approach to reprogramming the TME (Figure 1 and Table 3).

**Table 3:** Tumor microenvironment modulation strategies.

Strategy	Mechanism	Examples	Cancer indications
IDO1 inhibition	Reduces tryptophan metabolism and kynurenine‐mediated suppression	Epacadostat, BMS‐986205	Melanoma, RCC, pancreatic
TLR agonists	Stimulates innate immunity and antigen presentation via NF‐κB/IRF	Imiquimod, resiquimod, pixatimod	Melanoma, breast, lung
IL‐2 engineering	Enhances Teff and NK cell expansion while sparing Tregs	Bempegaldesleukin, DAB389IL2	Melanoma, RCC
Vitamin D supplementation	Modulates TME via microbiota, VDR signaling, and cytokine suppression	Calcitriol, vitamin D_3_	CRC, prostate, breast
CD73 blockade	Inhibits adenosine production and Teff cell suppression	Oleclumab	NSCLC, breast
Microbiota modulation	Alters immune tone, TME inflammation, and therapy responsiveness	FMT, probiotics	Pancreatic, melanoma, CRC
Chronotherapy	Aligns treatment timing with circadian biology to enhance efficacy	Time‐based ICI regimens	Under evaluation
Synthetic biology	Precision delivery of immune modulators using engineered platforms	Nanocarriers, oncolytic viruses	Broad application

*Note:* NF‐κB, nuclear factor kappa‐light‐chain‐enhancer of activated B cells; NK, natural killer cell.

Abbreviations: CRC, colorectal cancer; FMT, fecal microbiota transplantation; ICI, immune checkpoint inhibitor; IDO1, indoleamine 2,3‐dioxygenase 1; IL‐2, interleukin‐2; IRF, interferon regulatory factor; NSCLC, non‐small cell lung cancer; RCC, renal cell carcinoma; Teff, effectorT cell; TLR, toll‐like receptor; TME, tumor microenvironment; Treg, regulatory T cell; VDR, vitamin D receptor.

### 4.1. Indoleamine 2,3‐Dioxygenase 1

IDO1 guides the tryptophan degradation along the kynurenine pathway, leading to the production of metabolites that suppress Teff cell responses and promote Treg differentiation [159]. These immunosuppressive effects are further amplified by upregulation of PD‐1 and PD‐L1 [160]. IDO1 is overexpressed in response to the IFN‐γ pro‐inflammatory cytokine and is broadly expressed in tumor cells, DCs, macrophages, and endothelial cells, making it a versatile therapeutic target [161, 162]. Elevated IDO1 levels have been documented in multiple cancers, including melanoma, ovarian cancer, CRC, RCC, chronic lymphocytic leukemia, and sarcomas, and are associated with adverse outcomes and resistance to chemotherapy [163, 164]. Although preclinical studies provided strong support for combining IDO1 inhibition with PD‐1 or CTLA‐4 blockade [165], clinical translation has yielded heterogeneous results. A pivotal phase III randomized study evaluating the IDO1 inhibitor epacadostat in combination with PD‐1 blockade in melanoma failed to meet its primary clinical end points, prompting a critical reassessment of IDO1‐targeted therapeutic strategies [166]. Despite this setback, multiple IDO1 inhibitors remain under active clinical investigation. Agents such as BMS‐986205 and indoximod have been evaluated in combination with ICIs and chemotherapy across several tumor types, while additional compounds, including linrodostat and INCB024360, continue to be explored in ongoing clinical development programs [167–169].

### 4.2. TLRs and Innate Immune Activation

TLRs are pattern recognition receptors essential for innate immunity, detecting pathogen‐associated molecular patterns (PAMPs) and damage‐associated molecular patterns (DAMPs) to activate immune responses [170]. While some TLRs (e.g., TLR3 and TLR7–9) reside within endosomes, others (e.g., TLR2 and TLR4) are expressed on the cell surface. Ligand binding leads to dimerization and recruitment of the MyD88 or TRIF adaptor proteins, triggering downstream activation of NF‐κB, IRFs, and MAPKs [171, 172]. This cascade results in the production of the IL‐6, IL‐12, and TNF‐α pro‐inflammatory cytokines, and the MCP‐1 chemokine [172]. TLRs are found in macrophages, DCs, epithelial cells, and stromal cells [173]. Within the TME, their functions are context‐dependent. TLR7, TLR8, and TLR9 can enhance antigen presentation and promote T cell–mediated immunity [174], whereas TLR4 may facilitate tumor progression by inducing chronic inflammation, upregulating PD‐L1, and expanding Tregs [175–177]. Nonetheless, TLR4 can also exhibit antitumor effects by reprogramming TAMs from an M2‐like state to a pro‐inflammatory M1 phenotype, promoting cytotoxicity and antigen presentation [178]. TLR signaling can further induce autophagy and apoptosis in tumor cells, but the dual nature of these pathways presents challenges in therapy design [179].

Numerous TLR agonists are currently in clinical development. TLR7/8 dual agonists like telratolimod and TLR9 agonists such as pixatimod have shown early promise in Phase I trials [180, 181]. Other candidates include imiquimod (TLR7), resiquimod (TLR7/8), CPG‐52852 (TLR9), EMD‐1201081, agatolimod, rintatolimod, and motolimod, reflecting the broad interest in exploiting TLR signaling [182, 183]. Among these, imiquimod is already approved for treating superficial basal cell carcinoma and melanoma, working through localized activation of cytotoxic T cells [184]. TLR9 agonists, particularly CpG oligodeoxynucleotides, have demonstrated antitumor effects by enhancing DC maturation and promoting T cell activation in preclinical and clinical studies [185]. Combining TLR agonists with ICIs represents an emerging strategy for overcoming resistance in immunologically “cold” tumors [186]. Moreover, TLR ligands are being incorporated into cancer vaccine platforms to boost immunogenicity. For instance, monophosphoryl lipid A (MPLA), a TLR4 agonist, is an adjuvant in therapeutic vaccines, and resiquimod (R848) has shown efficacy in breast and lung cancer models by increasing TILs [187, 188].

### 4.3. Interleukin 2 and Its Receptors

IL‐2, a cytokine with a molecular weight of approximately 15 kDa, is enrolled in modulating immune responses and has been instrumental in the evolution of cancer immunotherapy [189]. IL‐2 mediates its effects through binding to two main types of receptors that differ in affinity and cellular expression. The high‐affinity IL‐2 receptor (IL‐2Rαβγ), composed of CD25 (IL‐2Rα), CD122 (IL‐2Rβ), and the common γ chain (CD132), is primarily expressed by Tregs. In contrast, the intermediate‐affinity receptor (IL‐2Rβγ), lacking the α subunit, is found on activated Teff and NK cells [190]. Upon receptor engagement, IL‐2 promotes the expansion and activation of Teff and NK cells, a mechanism that underpins its clinical utility. High‐dose IL‐2 therapy, such as aldesleukin, received approval in the 1990s for treating metastatic melanoma and RCC [191]. However, its application has been restricted due to side effects, including vascular leak syndrome, pulmonary edema, and hypotension, and its tendency to preferentially expand immunosuppressive Tregs [192]. To mitigate these issues, new therapeutic strategies have been developed to selectively stimulate the IL‐2Rβγ complex while avoiding IL‐2Rα engagement, thereby enhancing antitumor immune responses without amplifying Treg activity. Among these advances, bempegaldesleukin, a PEGylated IL‐2Rβγ‐selective agonist, has shown potential in early‐phase trials, demonstrating improved safety profiles and increased infiltration of immune effector cells in melanoma, NSCLC, and breast cancer [193]. Other innovative approaches include nanoparticle‐conjugated IL‐2 formulations, designed to improve delivery specificity and reduce systemic toxicity [193]. Additionally, fusion proteins like DAB389IL2, which couples IL‐2 to diphtheria toxin, enable targeted killing of IL‐2R‐expressing tumor cells while sparing normal tissues [194]. These engineered agents are also being evaluated in combination with ICIs, including nivolumab, to enhance therapeutic synergy [195]. Ongoing research is exploring alternative IL‐2–based agents such as denileukin diftitox, basiliximab, and daclizumab. These efforts aim to refine IL‐2 immunostimulation by minimizing toxicity while preserving or enhancing antitumor efficacy, offering new hope for overcoming the immunosuppressive barriers within the TME.

### 4.4. Vitamin D

Available through both diet (as D_2_ and D_3_) and endogenous production via UVB radiation, vitamin D_3_ is metabolized in the liver to form 25‐hydroxyvitamin D (25‐OHD), the standard biomarker for vitamin D sufficiency [196, 197]. Beyond its classical functions in bone and mineral physiology [198], vitamin D is increasingly being studied for its immunomodulatory and antitumor effects.

Experimental models have shown that vitamin D exerts antitumor effects by inhibiting cell proliferation, inducing apoptosis, and suppressing the tumor‐promoting functions of cancer‐associated fibroblasts, especially in melanoma and prostate cancer models [199]. Its immunomodulatory actions are mediated in part through the vitamin D–binding protein (Gc globulin), which regulates 25‐OHD bioavailability. A study by Giampazolias et al. [200] revealed that mice deficient in Gc protein (Gc^−^/^−^) showed greater tumor control and higher infiltration of activated CD4^+^ and CD8^+^ T cells compared to Gc^+^/^+^ counterparts. This immune enhancement was attributed to increased tissue availability of vitamin D, rather than its retention in the circulation. Moreover, the beneficial phenotype observed in Gc^−^/^−^ mice could be transferred to Gc^+^/^+^ mice through co‐housing, implicating gut microbial factors in mediating the response. Fecal microbiota transplantation (FMT) further confirmed that alterations in microbial communities, potentially involving vitamin D–regulated taxa like *Bacteroides fragilis*, contributed to enhanced antitumor immunity [200, 201].

Human data reinforce these findings. Higher serum levels of 25‐OHD have been linked to reduced incidence and improved outcomes in colorectal, breast, prostate, and pancreatic cancers [202]. In a clinical cohort from Granada, Spain, daily supplementation with 800 IU of vitamin D significantly lowered colorectal cancer risk, potentially by modulating inflammatory and proliferative biomarkers such as C‐reactive protein, Ki‐67, p21, p27, interleukins, β‐catenin, and DNA methylation profiles [203]. Beyond systemic effects, vitamin D also shapes the gut microbiota and supports mucosal immune homeostasis, which may enhance therapeutic responses across cancer types [204]. Expression levels of the vitamin D receptor (VDR) inversely correlate with tumor aggressiveness in several cancers, including ovarian cancer. VDR activation inhibits tumor invasion and metastasis, suggesting it may serve as a viable therapeutic target [205]. Furthermore, polymorphisms in the VDR gene influence both cancer susceptibility and the effectiveness of vitamin D supplementation strategies [206]. Calcitriol, the hormonally active form of vitamin D, has demonstrated synergy with chemotherapeutic agents, particularly in prostate cancer models, where it may help overcome resistance mechanisms and sensitize tumors to therapy [207].

## 5. Integrative Biomarker Platforms to Guide Next‐Generation Immune Checkpoint and TME–Targeted Therapies

The marked heterogeneity in patient responses to ICIs highlights the need for precision frameworks capable of capturing and integrating the full complexity of tumor‐immune biology. Multiomic profiling, spanning genomics, transcriptomics, epigenomics, proteomics, metabolomics, and spatial immune architecture, has emerged as an essential approach to map the dynamic interactions between cancer cells and the immune microenvironment with high resolution. Artificial intelligence (AI) and machine learning (ML) further strengthen this paradigm by enabling the integration of large‐scale, multidimensional datasets to reveal composite biomarkers, decode mechanisms of primary and acquired resistance, and optimize therapeutic decision‐making [208–211]. Evidence from AI–driven multiomics analyses consistently shows that integrated molecular signatures provide superior predictive power compared with traditional single‐parameter indicators such as PD‐L1 expression or TMB, underscoring the transformative potential of computationally guided immuno‐oncology [212].

Multiomic integration supported by ML enables a deeper and more granular understanding of immune phenotypes and their clinical relevance. Evidence from advanced multiomic analyses [213] shows that latent molecular programs, such as interferon‐driven inflammatory states, stromal remodeling trajectories, and tumor‐associated metabolic rewiring, stratify patients into biologically meaningful subgroups with distinct sensitivity to checkpoint blockade. Complementary AI–driven precision oncology frameworks. Shao et al. [214] demonstrate that deep learning models can simultaneously analyze mutational signatures, transcriptional networks, metabolic profiles, and clinicopathological variables to deliver individualized immunotherapy predictions with higher accuracy than traditional biomarkers. These integrative approaches align with contemporary systems biology methodologies, where graph‐based modeling and network‐level simulations resolve the architecture of immune regulatory circuits within the TME. Dynamic network maps exemplified by systems‐level immunology platforms such as those available at mechanistic insight into how perturbing specific checkpoints, cytokine nodes, or metabolic pathways may reprogram antitumor immunity [215].

AI–enabled prediction models are increasingly showing tangible clinical value across multiple tumor types. A multiomics framework for anti‐PD‐1 response prediction in melanoma has demonstrated superior performance over classical biomarkers by integrating exomic and transcriptomic features that capture both tumor‐intrinsic properties and immune microenvironmental signals [216]. Noninvasive strategies are also advancing rapidly. A deep learning radiomics model for HCC accurately predicted immunotherapy outcomes using contrast‐enhanced CT data, providing a valuable alternative for patients in whom tumor tissue is scarce or inaccessible [217]. In biliary tract cancers, combining bile metabolomics with immune signatures enabled high‐resolution stratification of PD‐1 responsiveness, illustrating the promise of metabolic‐immune integration for early therapeutic decision‐making [218].

AI is also reshaping the management of immune‐related toxicities. ML algorithms trained on real‐world melanoma cohorts have achieved clinically relevant accuracy in predicting irAEs, supporting proactive surveillance and risk‐adapted care. Digital health platforms that incorporate electronic patient‐reported outcomes further enhance this capacity; real‐time ML–assisted symptom monitoring enables earlier detection of emerging toxicities and facilitates timely clinical intervention. Integrating these tools into routine practice helps build safer and more adaptive immunotherapy regimens [219, 220].

Beyond prediction and safety, AI is accelerating the discovery of next‐generation immunomodulators. Integrated pipelines that combine ML–driven molecular modeling with systems biology simulations enable the rational design of small molecules targeting immune checkpoints, metabolic nodes, and stromal pathways. A representative example showcases an AI‐guided workflow that incorporates virtual screening, generative chemistry, and ADMET optimization to develop selective immune‐active compounds with reduced toxicity liabilities [221].

## 6. Conclusions and Future Perspectives

Immunotherapy has redefined the standard of care in oncology by leveraging the immune system’s intrinsic capacity to detect and eliminate malignant cells. ICIs targeting CTLA‐4 and PD‐1/PD‐L1 have produced durable responses and meaningful survival gains across multiple tumor types; however, only a subset of patients derive long‐term benefit, and many eventually develop resistance. Immune evasion, intratumoral and interpatient heterogeneity, and an immunosuppressive TME remain major barriers to efficacy. To overcome these limitations, the field is rapidly advancing toward next‐generation strategies that extend beyond classical checkpoints, including co‐inhibitory and co‐stimulatory modulators, bispecific antibodies, adoptive cell therapies, cytokine‐based treatments, therapeutic cancer vaccines, and synthetic immune modulators [222, 223].

The successful deployment of these novel interventions will depend on robust precision immunotherapy frameworks. Biomarker‐guided strategies integrating TMB, MSI status, T cell infiltration, TGF‐β pathway activity, prior treatment history, and proliferative indices are essential to refine patient selection, anticipate resistance, and guide rational combination regimens [6]. Moving beyond single‐parameter predictors, multiomic profiling will be required to construct composite biomarkers that more accurately capture tumor–immune dynamics and TME states. The integration of these multilayered datasets into clinically actionable decision tools is poised to enable personalized immunotherapeutic regimens that maximize efficacy while constraining toxicity [210, 224, 225].

As immunotherapy combinations become more potent and complex, the frequency and severity of irAEs are increasing, with toxicities affecting virtually every organ system and, in some cases, resulting in chronic morbidity [57]. Current management remains largely reactive, relying on early detection, high‐dose corticosteroids, and multidisciplinary care to prevent life‐threatening complications [226]. Future efforts must prioritize proactive risk stratification through predictive biomarkers of irAE susceptibility, elucidation of mechanisms that uncouple antitumor immunity from systemic autoimmunity, and the design of next‐generation immunomodulators with built‐in safety features. In parallel, standardized, globally applicable algorithms for toxicity monitoring and management will be critical to ensure safe implementation of advanced combination regimens in routine clinical practice.

The next phase of cancer immunotherapy will be driven by the convergence of mechanistic immunology, translational science, and technological innovation. AI and systems immunology will be central to this evolution, enabling predictive modeling of tumor–immune interactions, in silico testing of combination strategies, and real‐time adaptation of treatment based on longitudinal clinical and molecular data. These computational frameworks, combined with advances in nanomedicine, synthetic biology, and bioengineering, will improve the precision, delivery, and controllability of immunotherapeutic agents [227].

Equity in immunotherapy research and implementation is an urgent, cross‐cutting priority. The underrepresentation of non‐Caucasian populations in immunogenomic and clinical datasets undermines the development of universally applicable biomarkers, obscures population‐specific toxicity and response profiles, and risks widening existing global cancer disparities [228–230]. Addressing these inequities will require deliberate inclusion of diverse populations in clinical trials, expansion of immunogenomic efforts in low‐ and middle‐income countries, and context‐appropriate deployment of biomarker technologies. Only through globally representative data and infrastructure can precision immuno‐oncology become genuinely universal rather than a privilege restricted to select regions or ethnic groups.

Emerging areas of research are beginning to redefine how, when, and in whom immunotherapies are deployed. The timing, sequencing, and intensity of combination regimens are likely to be as important as the agents themselves; early, strategically designed combinations may preempt clonal escape and prevent the establishment of deeply entrenched immunosuppressive niches. The gut microbiome has emerged as a critical determinant of both therapeutic efficacy and irAE risk, with interventions such as FMT, targeted probiotics, prebiotics, and dietary modulation offering powerful levers to reprogram systemic immunity and the TME [231–234]. In parallel, circadian biology is gaining recognition as a key modulator of immune responses and drug pharmacodynamics. Chronotherapy, aligning the administration of ICIs and adjunct agents with intrinsic biological rhythms, together with circadian rhythm profiling, may enhance therapeutic indices by synchronizing immune activation and tissue susceptibility [235–238].

In conclusion, the future of cancer immunotherapy lies in an integrated, precision‐oriented paradigm that unites next‐generation immune targets, TME–modulating strategies, dynamic biomarker platforms, multiomic technologies, and cutting‐edge computational and bioengineering tools. By systematically addressing therapeutic resistance, immune‐related toxicity, and structural inequities in research and care delivery, immunotherapy can progress from a transformative option for selected patients to a broadly accessible, potentially curative modality that reshapes cancer outcomes worldwide.

## Author Contributions

Andrés López‐Cortés conceived the subject, contributed to the conceptualization of the study, supervised and did funding acquisition. All authors did data curation and gave conceptual advice and valuable scientific input. Finally, all authors wrote, reviewed, and edited the manuscript.

## Funding

This work was supported by Universidad de Las Américas, Quito, Ecuador.

## Disclosure

The authors have read and approved the submitted version.

## Ethics Statement

The authors have nothing to report.

## Consent

The authors have nothing to report.

## Conflicts of Interest

The authors declare no conflicts of interest.

## Data Availability

The authors have nothing to report.
